# Flood frequency analysis of Panchganga river basin using Gumbel and Log-Pearson Type III models

**DOI:** 10.1038/s41598-026-45840-3

**Published:** 2026-04-09

**Authors:** Sandipan Das, Amol Jarag, Rohit Bandivadekar, Pravin Parkar, Ketan Bardaskar, Shahista Inamdar

**Affiliations:** 1 Symbiosis International (Deemed University), Symbiosis Institute of Geoinformatics, Pune, Maharashtra 411016 India; 2 Department of Geography, Shikshanmaharshi Dr. Bapuji Salunkhe College, Miraj, Maharashtra 416410 India; 3 Department of Water Resources, Government of Maharashtra, Maharashtra, 416003 India; 4 Department of Statistics, Shikhanmaharshi Dr Bapuji Salunkhe College, Miraj, Maharashtra 416410 India

**Keywords:** Flood frequency analysis, GEV, LPT-III, Return periods, Panchganga river, Kolhapur, Climate sciences, Environmental sciences, Hydrology, Natural hazards

## Abstract

Floods are among the most frequent and destructive hydro-climatic hazards worldwide, causing severe damage to human life, infrastructure, and natural ecosystems. Flood Frequency Analysis (FFA) is a basic hydrologic analysis technique that estimates peak flood discharges and associated return periods, which are useful for an evidence-based understanding of flood risk and for flood mitigation planning. This study analyzed the Panchganga river basin in Maharashtra, India, known for recurring severe flood events due to intense monsoon rainfall and sediment-induced changes in river morphology. The analysis examines historical flood characteristics and forecasts potential flood magnitudes using the Gumbel Extreme Value Distribution and Log-Pearson Type III techniques. The Gumbel and Log-Pearson Type III distributions were selected due to their robustness, suitability for annual maximum flood series with moderate skewness, and continued relevance in design-oriented flood estimation for data-limited river basins. For this purpose, 43 years (1979–2021) maximum flood discharge per year data obtained from the Central Water Commission (CWC) and Maharashtra Water Resources Department (MWRD) were used. Return periods for flood magnitudes were estimated at intervals of 2, 5, 10, 20, 50, 100, and 200 years. The results indicate the highest recorded flood discharge of 4787.89 m^3^/s in 2019 and the lowest discharge of 725.26 m^3^/s in 2003. The 2-year flood event has a 50% possibility of occurrence in any year with average impacts, while severe flooding events are predicted at longer return periods, with discharge values exceeding the river’s carrying capacity. These findings will serve as a critical reference for policymakers and planners in formulating effective flood mitigation strategies, infrastructure resilience planning, and implementing early warning systems.

## Introduction

Floods are among the most widespread and destructive natural hazards worldwide, causing substantial loss of life and severe damage to infrastructure and human settlements^1^. As a climate-related hazard, flooding is a recurring phenomenon in fluvial environments, and nearly all river basins across the globe are vulnerable to flood events to varying degrees^2^. Hydrologically, floods occur when the discharge of a river, lake, or other water body exceeds its storage or channel capacity, resulting in the overflow of water onto adjacent floodplains^3^.

Flood processes are inherently complex and influenced by multiple interrelated factors, including rainfall intensity and duration, catchment characteristics, land use patterns, and channel morphology. Due to this complexity, accurate modelling and prediction of flood events remains challenging using purely analytical approaches^4^. Floods are considered the most frequent and economically damaging hydro-meteorological hazards, often producing high-intensity impacts on societies and economies^5^. While prolonged and intense rainfall is the primary trigger, flooding may also result from non-meteorological factors such as storm surges, rapid snowmelt, dam failures, and unplanned urban expansion^6^. In simple terms, flooding refers to the temporary inundation of areas that are normally dry.

India, characterized by a tropical monsoon climate, receives an average annual rainfall exceeding 1150 mm^7^. The strong seasonality and spatial variability of monsoon precipitation make many regions highly susceptible to hydrological extremes. Although heavy rainfall remains the dominant cause, additional factors such as storm surges, alterations in geomorphology, and anthropogenic modifications of river basins further intensify flood hazards^8^. Several major Indian river basins experience recurrent floods and droughts, reflecting the pronounced heterogeneity of hydrological regimes across the country^9^. Ultimately, regardless of the immediate triggering mechanism, flood occurrence is closely linked to atmospheric processes that regulate precipitation patterns and broader hydrological responses under changing climatic conditions^10^.

Flood Frequency Analysis (FFA) is a statistical method used to estimate the magnitude and probability of extreme flood events based on historical discharge records. It establishes a relationship between flood magnitude and recurrence interval, enabling the estimation of design floods for engineering and risk management purposes^11,12^. Numerous probability distributions have been applied in FFA, including Gumbel, Log-Pearson Type III (LP-III), Generalised Extreme Value (GEV), Weibull, and Log-Normal distributions. Studies across the USA, Australia, Europe, and Asia have evaluated their performance under different hydro-climatic conditions. Similar frequency-based approaches have also been applied to other natural hazards such as drought analysis and bushfire risk assessment, highlighting the broader applicability of extreme-value theory in environmental hazard studies.

Based on historical time series of flow and gauge levels, FFA is a significant hydrologic and fluvial geomorphometric technique used for forecasting the intensity and frequency of floods over a certain recurrence interval^11^. The quality of FFA depends on the choice of a suitable frequency distribution of the data, wrong choice may result in large errors and bias in flood estimate^12^. FFA plays a vital role in hydrologic studies, particularly in understanding flood recurrence patterns which are directly linked to infrastructure planning and economic activities^13^. Engineers require the flood return period for the design of reservoir barriers, Drainage conduits, Embankments, bridge, major roadways, and manufacturing zones structures to manage risks and prevent costly damage^14^. The main aim of FFA is to predict the recurrence interval of a given inundation event. Prior to estimating design flows, the streamflow data must be analysed to derive the most suitable probability distribution for flood events^15^. This technique can give you a sense of how often a river might achieve critical water elevations, which is helpful for researchers trying to discern past and long-term river behavior. The relationship between floods of different magnitudes and their occurrence is also important—that is, small floods are frequent, large floods are rare but damaging^16^. FFA is dependent upon historic or synthetic estimated distribution of annual maximum peak discharges recorded by gauging stations to determine model parameters and provide estimates of extreme flood events at long return periods^17^. Accurate flood frequency estimates are vital for the management of floodplains, protection of human life, reduction in the cost of flooding, design of structures for flood control, and evaluation of the hazard of floods. In effect, FFA facilitates probability estimation of flooding at land/scale and plays a crucial part of managing the risk of flooding and infrastructure resilience decision support.

Particularly with respect to climate change and the increasingly regular extreme weather, flood research has seen a notable growing in the last few years. Seasonal environmental characteristics can affect flood hazard when the wetlands in the flood-plains are most affected by main river processes (discharge, channel changes and flood events). Advanced techniques such as Fuzzy Logic are increasingly being used to evaluate flood risks in sensitive ecosystems to address these risks^18^. Utilization of such remote sensing-based inundation maps is of great significance of assessing the past as well as presence states of wetlands and waters. These maps are useful for analyzing the environmental to logical consequences of climate change and natural hydrological processes^19^. Flood Frequency Analysis (FFA) is also getting importance in dealing with extreme climatic conditions. The extreme value theory (EVT) has been used to gain an understanding on flood and cyclone-specific vulnerability and risk assessments in Indian sub-continent^20^.

Using FFA, one can quantify both the probability of flood occurrence and the expected severity of such events in riverine environments. Projected frequency distribution diagrams offer an outlook on possible future flood occurrences^12^ and are based on descriptive statistical measures such as arithmetic average (x̄), Measure of dispersion (SD), and Asymmetry of the distribution (Sk) that are computed by FFA from observed annual maximum discharge data. Techniques such as L-moment analysis and Gumbel’s distribution are important in FFA. They alleviate the potential of bias and outliers in flood data sets and enhance flood prediction precision^21^. Additionally, FFA allows to understand both the re-occurrence of floods and their magnitudes. It is also important for the detection of scenarios with the most damaging flooding effects. It’s by looking at historical records of floods that scientists can anticipate whether an area is likely to get flooded. Such analyses are important for infrastructure planning for flood prevention, incorporating hydraulic structures such as dams and bridges. Data-driven methods have been used in different ways to reveal flood patterns, increase the accuracy of flood forecast, and decrease the uncertainty. Such solutions assist in the evaluation of effects of parameters, say, rainfall intensity and river flow, in the flooding issue^15^.

In recent years, machine learning (ML) and deep learning (DL) approaches have gained increasing attention in flood frequency analysis (FFA) and flood hazard modelling at the global scale^22,23^. In Europe, Toth, Brath, and Montanari^24^ applied artificial neural networks for short-term rainfall–runoff forecasting in the Reno River basin of the Apennines Mountains, Italy. Algorithmic modelling techniques, including Artificial Neural Networks (ANN), Support Vector Regression (SVR), Adaptive Neuro-Fuzzy Inference Systems (ANFIS), Random Forest (RF), and other neural computing frameworks, have been widely applied to capture the nonlinear relationships between hydrometeorological variables and flood responses^25,26^. For example, Shahiri and Afzalimehr^27^ applied ANN, SVR, and ANFIS models in combination with GIS-based techniques to simulate flood-prone areas in Mazandaran Province, Iran, demonstrating the capability of data-driven approaches to represent complex spatial patterns. Recent studies further highlight that advanced ML frameworks can outperform conventional hydrological models in simulating runoff and extreme flood behaviour, particularly in complex urban catchments^28^.

Compared to conventional process-based hydrological models, which require detailed physical parameterization and extensive calibration, ML-based models are primarily data-driven and can efficiently learn nonlinear interactions among rainfall, discharge, catchment characteristics, and land-use variables without requiring explicit assumptions about underlying physical processes. Numerous ML algorithms, including Artificial Neural Networks (ANN), Support Vector Machines (SVM), Random Forest (RF), Gradient Boosting, and deep learning architectures, have been successfully applied in flood forecasting studies. Several comparative investigations have reported that ML-based models often achieve higher predictive accuracy and improved performance metrics (e.g., lower RMSE and higher R^2^ or NSE values) compared to traditional conceptual or physically based hydrological models, particularly in complex and data-rich catchments^28^.

This flexibility enables ML models to perform effectively in basins where physical parameters are uncertain, highly variable, or difficult to quantify. Furthermore, ML approaches can accommodate high-dimensional datasets and integrate diverse inputs, including remote sensing products, climatic indices, and land-use variables, thereby improving predictive robustness and generalization capability. Ensemble modelling strategies, which combine multiple ML algorithms or integrate ML techniques with traditional statistical methods, have further enhanced flood estimation accuracy and reduced prediction uncertainty. Similarly, the integration of Fuzzy Logic with GIS-based spatial analysis has been shown to improve flood susceptibility mapping and hazard assessment by effectively addressing uncertainty and imprecision in environmental variables^29^. Collectively, these studies demonstrate that data-driven and hybrid modelling approaches provide a robust complementary framework to process-based models for flood frequency analysis and risk assessment.

Although Gumbel and Log-Pearson Type III (LP-III) distributions are well established in flood frequency analysis, widely adopted in national guidelines in several developed countries, their basin-specific performance in medium-sized, data-constrained river basins of India remains insufficiently explored. In particular, river systems undergoing rapid land-use transformation and experiencing increasing flood intensity require localized statistical evaluation rather than direct methodological transfer from other hydro-climatic regions. The Panchganga river basin has received limited systematic flood frequency assessment despite repeated extreme flood events in recent decades. This study addresses this gap by providing a long-term (43-year) flood frequency evaluation for the basin and assessing the suitability of LP-III and Gumbel distributions under moderate skewness conditions. The novelty of this research lies in establishing basin-specific design flood estimates, integrating hydrograph analysis and uncertainty assessment, and providing scientifically derived flood magnitudes to support regional flood management and infrastructure planning in an understudied yet highly flood-prone river system.

Floods of different magnitude (i.e., probability or return period) have a great deal of information concerning the time evolution of hydrological systems. The predicted time interval between comparable flows is called recurrence interval^11^. The Panchganga Basin is regularly experiencing floods, brought about by high volume monsoon rainfall, land use transformation, as well as alterations in river morphology. During last century, the course of the Panchganga River channel has been subject to profound changes mostly because of sedimentation, urban growth, and extreme hydrological events. According to the CWC and Maharashtra Water Resources Department (MWRD) hydrological data, the large flood events were observed from 1989, 2005, 2019 and 2021, in which severe water levels were found above the DML from Terwad, Ichalkaranji, and Kurundwad gauge stations^30^. The Panchganga River has been reported to be crossing the danger (548.5 m) level repeatedly on several occasions in Kurundwad, especially during the monsoon floods, causing the repeated inundation in the region. The basin is subjected to frequent floods during robust monsoons, decreased outflow capacity and human interference, squeezing rural and cities located around its path.

While the Generalised Extreme Value (GEV) distribution is widely recommended in contemporary flood studies and national guidelines such as Australian Rainfall and Runoff (2019), the present study adopts the Gumbel and Log-Pearson Type III distributions for practical and data-driven reasons. The available annual maximum discharge dataset for the Panchganga River exhibits moderate skewness (Cs ≈ 0.31), making LP-III statistically appropriate. Similar concerns regarding parameter sensitivity and data length influence have been reported in extreme value modelling studies^31–34^. Furthermore, Indian flood estimation practices continue to rely heavily on Gumbel and LP-III distributions due to their robustness, transparency, and suitability for limited datasets. The Gumbel distribution offers computational simplicity and ease of interpretation for engineering design, while LP-III effectively captures skewed flood behaviour. Therefore, the study prioritises methodological reliability and regional applicability over added model complexity.

This study concentrates on the FFA by employing the Gumbel extreme value method for LPT-III distribution in the Panchganga Basin, which is an inundation area and is less represented hydrologically. The study also used historical flood data from 1979 to 2021 to analyze flood recurrence intervals and obtain deeper insights into the flood characteristics of the less analyzed river system. Flood magnitudes were estimated for return periods of 2, 5, 10, 20, 50, 100, and 200 years to assess the probability and severity of flood occurrences in the basin. Quantitative forecasting of flood occurrences in terms of their frequency and magnitude within the Panchganga Basin plays a crucial role in informed flood hazard reduction and sustainable infrastructure planning. Furthermore, this study aims to be a vital tool for planners to develop mitigation plans, improve the urban drainage systems, and deploy sustainable flood defense against flooding with scientifically-based return period for flood events.

## Study area

**Fig. 1:**
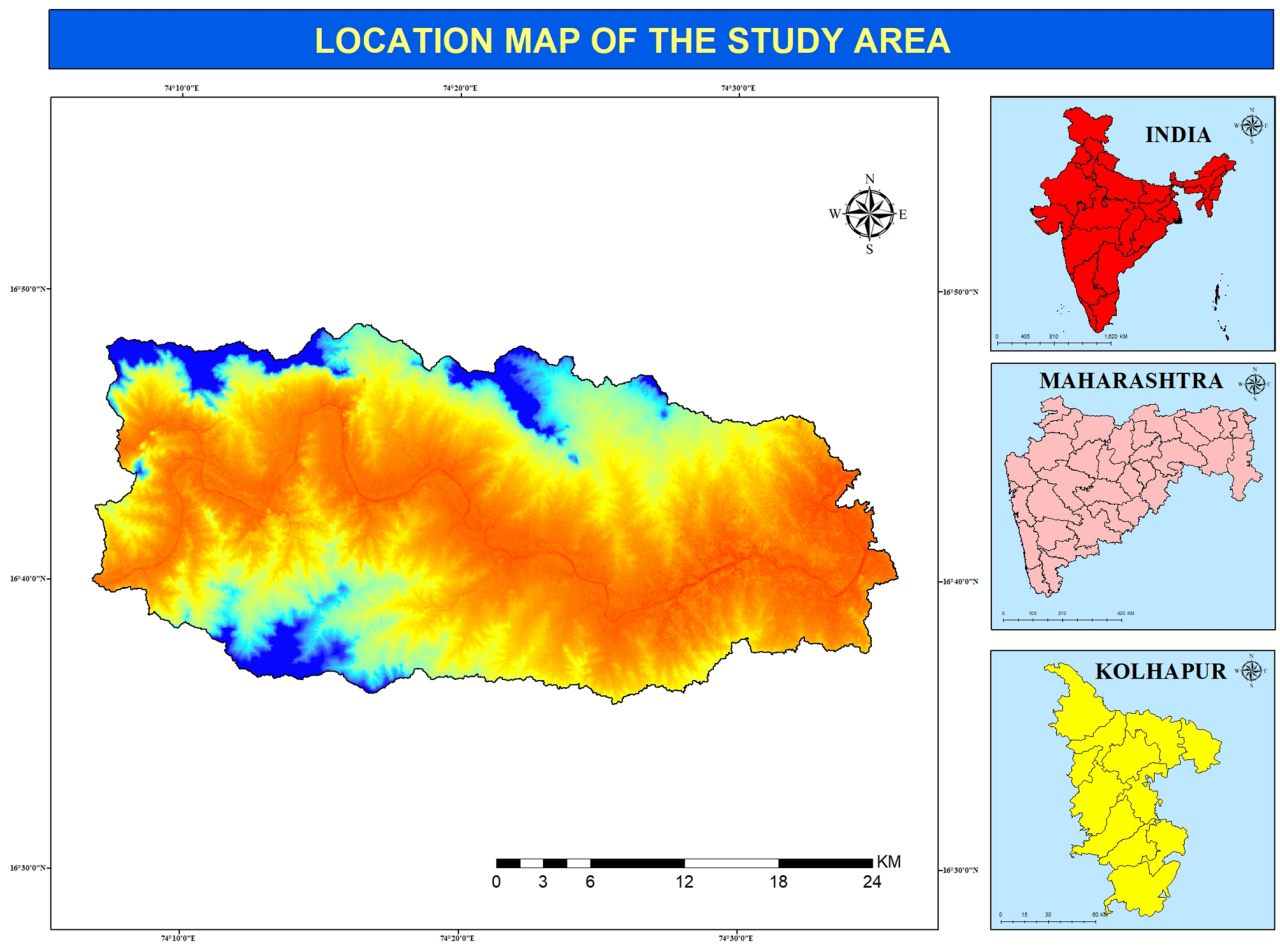
Location map of study area.

A significant tributary of the Krishna River, the Panchganga river basin (Fig. 1) meets its confluence close to Narsobawad. It lies between 16° 25′ north and 16° 55′ north latitudes and 74° 05′ east to 74° 30′ east longitude in northern Kolhapur district, Maharashtra. Unlike rivers with independent origins, it is generated at Prayag by the convergence of five tributaries, namely Kasari, Kumbhi, Tulsi, and Bhogawati, with a subterranean Saraswati stream thought to contribute. The basin, which covers 892 square kilometers, has a complicated geological structure, undulating physiography, and a diverse ecosystem. It receives yearly rainfall between 2500 mm and 3500 mm, generating a good climatic environment and making it an important hydrological system in the Kolhapur region.

## Methodology

The Central Water Commission (CWC) of the Indian government, and the Maharashtra Water Resources Department (MWRD) provide data on maximum annual discharge statistics for the Panchganga Basin that extend 43 years, from 1979 to 2021 (https://www.cwc.gov.in). These datasets provide comprehensive insights into flood patterns, recurrence intervals, and hydrological variability, enhancing flood hazard analysis and mitigation measures in the Panchganga river basin. For Flood Frequency Analysis (FFA), key statistical parameters such as mean, standard deviation, and Skewness are computed using 43 years (1979–2021) of historical peak discharge data for the Panchganga basin. A long-term dataset minimizes sampling errors and enhances the accuracy and reliability of flood predictions. FFA measures maximum flow at precise river sites within numerous return periods using Statistical distribution approaches, including gaussian and log-normal distributions. Choosing the most suitable probability function is critical, as discharge values are usually estimated using annul maximum flows recorded at a one monitoring station, assuming independent and identically distributed (IID) flows. Inaccurate recorded measurements can affect the findings since parameter estimation utilizes sample data. Incomplete time series or data gaps impede exact estimates. Reliable FFA usually calls for a minimum 30-year dataset; however, longer datasets enhance flood forecast accuracy and lower sampling error rates. Flood frequency estimation in this work uses the Log-Pearson Type III and Gumbel extreme value distributions.

### Gumbel extreme value distribution method (GEV)

The GEV is one well-liked statistical method for forecasting catastrophic floods and other extreme hydrological events. The basic equation for GEV is used to forecast the magnitude of the flood (X_T) for a particular return period (T) is:

1 $${\mathrm{X}}\_{\mathrm{T}} = \left( {{\overline{\mathrm{X}}} + {\mathrm{K}}} \right) \times {\mathrm{Sd}}$$

where Sd = Standard deviation of the sample size, K = Frequency factor, expressed as:

2 $${\mathrm{K}} = \left( {{\mathrm{Y}}\_{\mathrm{T}} - {\overline{\mathrm{Y}}}\_{\mathrm{n}}} \right)/{\mathrm{S}}\_{\mathrm{n}}$$

Y_T = Reduced variate, given by:

$${\mathrm{Y}}\_{\mathrm{T}} = - {\text{ ln }}\left( {{\text{ln }}\left( {{\mathrm{T}}/\left( {{\mathrm{T}} - {1}} \right)} \right)} \right)$$

X̄ = Mean of the sample data, Ȳ_n = Mean of reduced variate, S_n = Standard deviation of reduced variate, The parameters Ȳ_n and S_n are determined based on the sample size when using Gumbel’s Extreme Value Distribution.

The return period (T_r) in the Gumbel Extreme Value Distribution is calculated using the formula:

3 $${\mathrm{T}}\_{\mathrm{r}} = \left( {{\mathrm{n}} + {1}} \right)/{\mathrm{n}}$$

where n = Rank number of the flood discharge for a particular year (ranked from highest to lowest).

### Log Pearson type III distribution method (LPT-III)

The LPT- III distribution is one of the prevalent probability distributions used in flood frequency analysis (FFA) of runoff, especially for the type of data possessing Skewness. It is frequently applied in hydrological research for estimating flood peaks.

The formula of the LPT- III Distribution for calculating the amount of flood (X_T) in a certain return period (T) is:

4 $${\mathrm{X}}\_{\mathrm{T}} = \left( {{\overline{\mathrm{Z}}} + {\mathrm{K}}\_{\mathrm{s}}} \right) \times {\mathrm{Sd}}$$

Where Sd = Standard deviation of the sample size. K_s = Standard variate, obtained from statistical tables based on the skewness coefficient. Z̄ = Mean logarithm of the sample data.

The coefficient of Skewness (C_s) is determined using the formula:

5 $$C_{s} = N \times \sum Z\left( {Z - Z^{ - } } \right)^{3} /\left( {N - 1} \right) \times \left( {N - 2} \right) \times \left( {Sd} \right)^{3}$$

Where: C_s_ = Coefficient of Skewness, N = Number of years in discharge data, Σ (Z − Z̄)^3^ = Sum of the cube of deviations from the Mean,

The Antilog of Z_T or 10^(Z_T) represents the recurrence value of the flood, signifying the recurrent line of the Log Pearson Type III Distribution.

### Method for hydrograph analysis

A hydrograph is a plot time along the x-axis that shows rate of flow on the y -axis of a river channel. It is generally expressed in m^3^/s and displays the catchment’s reaction to the input of rainfall and may also indicate changes in stage, discharge, and velocity with time. Hydrographs are key information to analyze the seasonality of flood episodes and explain the hydrological performance of basins. This study generated an annual peak discharge hydrograph for the Panchganga basin based on recorded discharge data from 1979 to 2021. The generated hydrograph shows the flood wave changes during high-flow seasons, so capturing both natural flood peaks and human effects on river outflow. This analysis is essential for flood forecasting and regional water resource management.

### Uncertainty analysis method

The Standard Error (SE) is calculated to assess uncertainty in flood discharge data. In hydrological studies, SE quantifies the variation or potential variability due to with the mean flood discharge value, providing insight into data reliability. A smaller SE indicates higher confidence in the computed mean flood discharge, reducing the impact of data variability.

The SE is calculated using:

6 $${\mathrm{SE}} = \sigma /\surd {\mathrm{n}}$$

Where SE = Standard error. σ = Standard deviation of the dataset. n = Number of years in the dataset.

For this study, 43 years of flood discharge data (1979–2021) have been used, with a calculated SD value of 835.40. This calculation ensures robustness in flood frequency analysis, helping refine flood risk assessments and infrastructure planning.

## Results and discussion

We started by emphasizing the key findings the subsect could gather from the FFA for the Panchganga river. Distributions 2.3.1–2.3.4 show uncertainty, time of concentration, LPT-III, and Statistic GEV distribution. Then, a sequential table demonstrating the design flood estimates for various recurrence intervals, the recurrence interval calculation employing both methods, and the highest flood discharge from 1979 to 2021 follows (Table 1).

**Table 1:** Variance values for specific years.

Year	Standard variant
2	0
5	0.842
10	1.282
20	1.645
50	2.054
100	2.326
200	2.576

### Analysis of return period and expected peak water discharge estimation

The interval between occurrences in the Panchganga river basin is estimated for 2, 5, 10, 20, 50, 100, and 200 years using GEV distribution approach and the LPT-III probability distribution method. Expected hydrologic discharge in GEV distribution is 1887.93 m^3^/s with a SD of 835.40 m^3^/s. In LPT-III distribution, the expected hydrologic discharge is 3.2407 m^3^/s with a SD of 0.1735 m^3^/s. The study focused on the annual maximum flow series, the average (x) and (z), Statistical deviation (sd), and Measure of asymmetry (Cs) for the Logarithmically transformed data. The K for various return periods was also determined K for various return periods was from values of K = YT − Yn Sn Standard variants (or Ks) using the values of obtained from the K (Table 2) In the analysis, peak discharge data for the year 1979 to 2021, estimates RI (recurrence interval) of 2, 5, 10, 20, 50, 100, 200 years. The flow rate due to 10-year RI (recurrence interval) is found to exceed the river’s carrying capacity. This may occur because of the large amount of sediment washed down by the river, resulting in a raised streambed. Therefore, to mitigate the impact of flooding, the bed of the river has to be dredged and the embankment to be raised to prevent flood.

**Table 2:** Recurrence intervals were derived through the application of Gumbel’s Type I distribution for extreme value analysis.

Sl. No	Year	Peak flood discharge (m^3^/s) (x)	Rank order (n)	(x − x̄)	(x − x̄)^2^	Sx^2^ = (n − x̄)^2^	Return period (Tr = n + 1/m)
1	2019	4787.89	1	2941.894	8,654,740.0249	3,404,021.4648	44.00
2	2021	4100.00	2	2254.001	5,080,520.3189	3,400,332.4667	22.00
3	1997	3590.00	3	1744.001	3,041,539.3417	3,396,645.4686	14.67
4	2005	3340.00	4	1494.001	2,232,038.8627	3,392,960.4705	11.00
5	2006	2796.56	5	950.561	903,566.1350	3,389,277.4724	8.80
6	1994	2680.00	6	834.001	695,557.5980	3,385,596.4744	7.33
7	1991	2553.00	7	707.001	499,850.3547	3,381,917.4763	6.29
8	2016	2267.37	8	421.369	177,551.6257	3,378,240.4782	5.50
9	1990	2250.00	9	404.001	163,216.7741	3,374,565.4801	4.89
10	1989	2205.00	10	359.001	128,881.6879	3,370,892.4820	4.40
11	2020	2117.00	11	271.001	73,441.5193	3,367,221.4839	4.00
12	1988	2080.00	12	234.001	54,756.4484	3,363,552.4859	3.67
13	2018	2072.09	13	226.090	51,116.6454	3,359,885.4878	3.38
14	1986	2050.00	14	204.001	41,616.3909	3,356,220.4897	3.14
15	2008	1951.57	15	105.566	11,144.1715	3,352,557.4916	2.93
16	1980	1917.60	16	71.601	5126.6972	3,348,896.4935	2.75
17	1996	1900.00	17	54.001	2916.1035	3,345,237.4954	2.59
18	2013	1854.07	18	8.073	65.1682	3,341,580.4974	2.44
19	2004	1832.00	19	− 13.999	195.9732	3,337,925.4993	2.32
20	2007	1820.56	20	− 25.439	647.1449	3,334,272.5012	2.20
21	1984	1795.00	21	− 50.999	2600.9023	3,330,621.5031	2.10
22	1981	1790.00	22	− 55.999	3135.8927	3,326,972.5050	2.00
23	2011	1720.64	23	− 125.361	15,715.4266	3,323,325.5069	1.91
24	1985	1570.00	24	− 275.999	76,175.4712	3,319,680.5089	1.83
25	1983	1545.00	25	− 300.999	90,600.4233	3,316,037.5108	1.76
26	2017	1542.07	26	− 303.933	92,375.3674	3,312,396.5127	1.69
27	1999	1540.00	27	− 305.999	93,635.4137	3,308,757.5146	1.63
28	2014	1534.09	28	− 311.908	97,286.5192	3,305,120.5165	1.57
29	2010	1463.83	29	− 382.171	146,054.7053	3,301,485.5184	1.52
30	1993	1447.00	30	− 398.999	159,200.2355	3,297,852.5204	1.47
31	2002	1443.00	31	− 402.999	162,408.2278	3,294,221.5223	1.42
32	1982	1430.60	32	− 415.399	172,556.3641	3,290,592.5242	1.38
33	1987	1392.00	33	− 453.999	206,115.1301	3,286,965.5261	1.33
34	2009	1344.51	34	− 501.493	251,495.2711	3,283,340.5280	1.29
35	1992	1306.00	35	− 539.999	291,598.9653	3,279,717.5299	1.26
36	2015	1285.65	36	− 560.353	313,995.8054	3,276,096.5318	1.22
37	1995	1170.00	37	− 675.999	456,974.7047	3,272,477.5338	1.19
38	2001	1150.00	38	− 695.999	484,414.6664	3,268,860.5357	1.16
39	1998	1051.00	39	− 794.999	632,023.4767	3,265,245.5376	1.13
40	2012	1010.61	40	− 835.385	697,868.5246	3,261,632.5395	1.10
41	2000	890.00	41	− 955.999	913,934.1682	3,258,021.5414	1.07
42	1979	870.00	42	− 975.999	952,574.1299	3,254,412.5433	1.05
43	2003	725.26	43	− 1120.73	1,256,049.4251	3,250,805.5453	1.02

x = Peak flood discharge in cubic meters (m^3^/s), x̄ = Mean of the given sample. (which is 1887.93 m^3^/s), Sd = Standard Deviation of the given sample (which is 835.40 m^3^/s).

Table 3 lists the anticipated water flow for flood events corresponding to various recurrence intervals of two, five, ten, twenty, fifty, one hundred, and two hundred years, as well as the calculations for each. Gumbel’s predicted flood for the Panchganga river with a return duration is y = 6295.6(x) + 1333.9, based on an R2 of 0.8403. It is significant that GEV expected higher flood estimates for the 200-year period and lower forecasts for the 2-year period for the 2, 5, 10, 20, 50, 100, and 200 return periods. Ranging from two years to 1000 years, the study includes floods of different lengths and intensities. The return periods, peak flow rates (m^3^/s), and related flood magnitudes are summarized in Table 3.

**Table 3:** Gumbel’s distribution was employed to compute the expected flood magnitude.

Return period (T) (Years)	Reduced variate (Y_T) = − [Ln x Ln x { T/(T − 1)	Frequency factor (K) = (yT − yn)/Sn	Expected flood (XT) (m^3^/s) = x_bar K × Sd
2	0.3665	− 0.1557	1757.8254
5	1.4999	0.8315	2582.6230
10	2.2503	1.4852	3128.7108
25	3.1985	2.3111	3818.6941
50	3.9019	2.9239	4330.5632
100	4.6001	3.5320	4838.6527
200	5.2958	4.1380	5344.8883

**Fig. 2:**
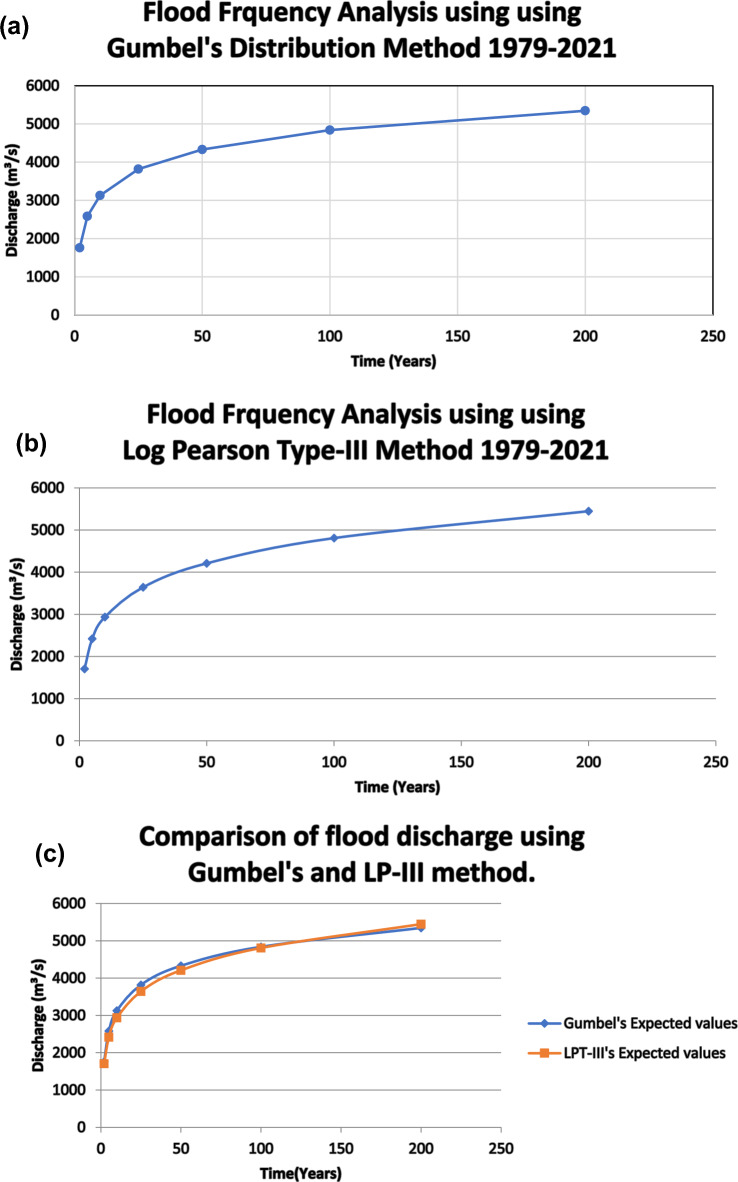
(**a**) FFA based on Gumbel’s distribution. (**b**) FFA based on Log-Pearson Type III distribution. (**c**) Comparison of Flood Discharge in Gumbel & Log-Pearson Type III distribution.

Figure 2a and b, as well as Tables 4 and 5, display the graphical representation of the GEV and LPT-III distribution. Both diagrams offer a clear understanding of the flood occurrence patterns within the study area. Annual floods come in the Panchganga river basin, and Fig. 2a and b show that maximum flood values estimated for different recurrence intervals of 2, 5, 10, 20, 50, 100, and 200 years rises as the FFA predicted using GEV and LPT- III distribution increases. Longer recurrence intervals (10, 50, 100, 500, and 1000 years) are expected to result in more severe floods, which will cause higher peak discharges and larger floods.

**Table 4:** Recurrence interval estimation based on the Log-Pearson Type III distribution.

Year	Discharge (m^3^/s)	Rank	Log discharge (Z)	(Z − Z̄)	(Z − Z̄)^2^	(Z − Z̄)^3^
2019	4787.89	1	3.6801	0.4394	0.1931	0.084847
2021	4100.00	2	3.6128	0.3721	0.1384	0.051503
1997	3590.00	3	3.5551	0.3144	0.0988	0.031068
2005	3340.00	4	3.5237	0.2830	0.0801	0.022670
2006	2796.56	5	3.4466	0.2059	0.0424	0.008729
1994	2680.00	6	3.4281	0.1874	0.0351	0.006582
1991	2553.00	7	3.4071	0.1663	0.0277	0.004601
2016	2267.37	8	3.3555	0.1148	0.0132	0.001513
1990	2250.00	9	3.3522	0.1115	0.0124	0.001385
1989	2205.00	10	3.3434	0.1027	0.0105	0.001083
2020	2117.00	11	3.3257	0.0850	0.0072	0.000614
1988	2080.00	12	3.3181	0.0773	0.0060	0.000463
2018	2072.09	13	3.3164	0.0757	0.0057	0.000434
1986	2050.00	14	3.3118	0.0710	0.0050	0.000358
2008	1951.57	15	3.2904	0.0497	0.0025	0.000122
1980	1917.60	16	3.2828	0.0420	0.0018	0.000074
1996	1900.00	17	3.2788	0.0380	0.0014	0.000055
2013	1854.07	18	3.2681	0.0274	0.0008	0.000021
2004	1832.00	19	3.2629	0.0222	0.0005	0.000011
2007	1820.56	20	3.2602	0.0195	0.0004	0.000007
1984	1795.00	21	3.2541	0.0133	0.0002	0.000002
1981	1790.00	22	3.2529	0.0121	0.0001	0.000002
2011	1720.64	23	3.2357	− 0.0050	0.0000	0.000000
1985	1570.00	24	3.1959	− 0.0448	0.0020	− 0.000090
1983	1545.00	25	3.1889	− 0.0518	0.0027	− 0.000139
2017	1542.07	26	3.1881	− 0.0526	0.0028	− 0.000146
1999	1540.00	27	3.1875	− 0.0532	0.0028	− 0.000151
2014	1534.09	28	3.1859	− 0.0549	0.0030	− 0.000165
2010	1463.83	29	3.1655	− 0.0752	0.0057	− 0.000426
1993	1447.00	30	3.1605	− 0.0803	0.0064	− 0.000517
2002	1443.00	31	3.1593	− 0.0815	0.0066	− 0.000541
1982	1430.60	32	3.1555	− 0.0852	0.0073	− 0.000619
1987	1392.00	33	3.1436	− 0.0971	0.0094	− 0.000915
2009	1344.51	34	3.1286	− 0.1122	0.0126	− 0.001411
1992	1306.00	35	3.1159	− 0.1248	0.0156	− 0.001943
2015	1285.65	36	3.1091	− 0.1316	0.0173	− 0.002279
1995	1170.00	37	3.0682	− 0.1725	0.0298	− 0.005136
2001	1150.00	38	3.0607	− 0.1800	0.0324	− 0.005835
1998	1051.00	39	3.0216	− 0.2191	0.0480	− 0.010521
2012	1010.61	40	3.0046	− 0.2361	0.0558	− 0.013168
2000	890.00	41	2.9494	− 0.2913	0.0849	− 0.024727
1979	870.00	42	2.9395	− 0.3012	0.0907	− 0.027327
2003	725.26	43	2.8605	− 0.3802	0.1446	− 0.054972

**Table 5:** Calculation of statistical estimates of expected flood magnitudes using the Log-Pearson Type III distribution.

Return period	Ks	ZT = z + Ks*Sd	Expected Flood Antilog of ZT = (10^ZT)
2	− 0.05	3.2320	1706.287
5	0.824	3.3836	2419.120
10	1.309	3.4677	2936.203
25	1.849	3.5614	3642.967
50	2.211	3.6242	4209.672
100	2.544	3.6820	4808.515
200	2.856	3.7361	5446.668

### Assessment of flood discharge variability based on GEV and LPT-III statistical models.

**Fig. 3:**
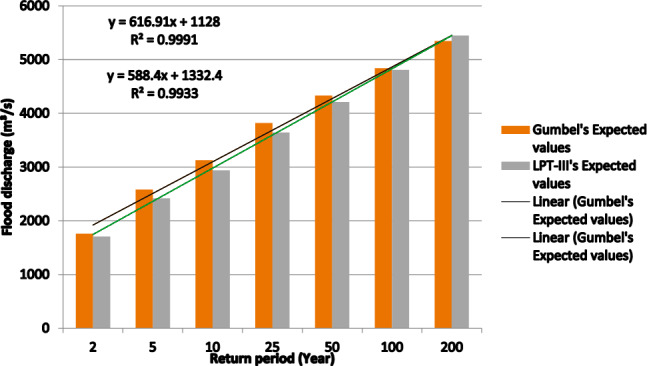
Comparison of flood discharges computed using LP-III and GEV methods.

**Fig. 4:**
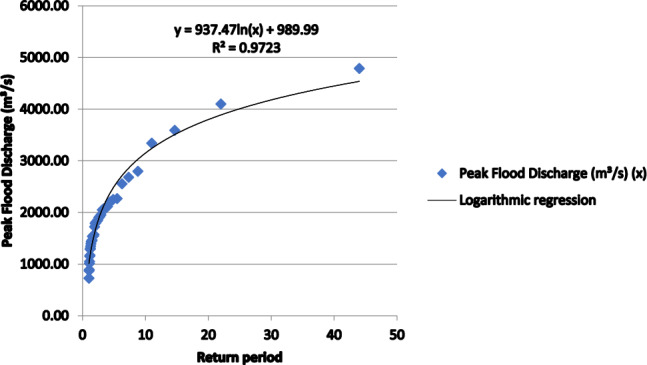
Different return periods of flood in the Panchganga River Basin from 1979 to 2021.

Both the Gumbel and LPT-III techniques are well-known for the flood frequency FFA (Fig. 3) and estimated extreme flows over several RI (recurrence intervals) (Fig. 4). The LPT-III distribution, which better represents extreme flood episodes (Fig. 2b) and considers Skewness in the data, is more appropriate for the Panchganga River, nevertheless.

Where, Z = Peak flood discharge in cubic meters (m^3^/s), z = Mean of the given sample. (which is 3.2407 m^3^/s), Sd = Standard Deviation of the given sample. (which is 0.1734 m^3^/s), Cs = Skewness coefficient of the given sample. (which is 0.3115).

Flood frequency studies often use the LPT-III and GEV distributions^12,13^. The nature of the data, though, will determine the distributional selection. Data with extensive variability and an associated skewness coefficient (Cs) near 0.3 (which is 0.3115) is better suited for the LPT-III distribution. Furthermore, the 1979–2021 dataset reveals extreme occurrences with more variability, which fits the LP-III approach. The LPT- III distribution is suitable since the skewness coefficient (Cs) is around 1, suggesting modest data variability. Given the growing propensity of peak discharges for longer return periods, sufficient flood management policies are even more vital. Data collected underlined the possibility of catastrophic floods and the necessity of proactive strategies and well-prepared infrastructure in flood-prone areas such the valley of the Panchganga river basin.

### Using hydrograph data to predict future flood levels and examine potential flood changes

**Fig. 5:**
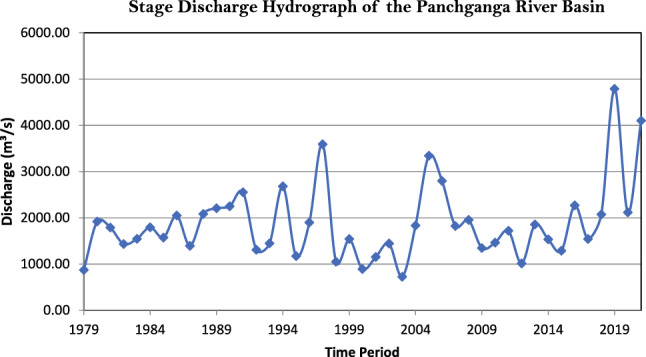
Stage discharge hydrograph of Panchganga river basin.

Heavy monsoon rains in 2019 caused a notable peak, with a total discharge noted of 4748 m3/s (Fig. 5). Heavy rains often fall in the area throughout the rainy season, hence raising river flows. Moreover, the alterations of water flow system due to the land-use variations (deforestation, urban expansion) might also result in change of capacity for water transport of this Panchganga river basin. Also, the basin topographic and soil structure of the basin could also influence water infiltration and movement within the basin. Understanding these interactions is essential for optimum management of hydrological resources and to minimize the effect of major incidents, such as floods. The minimum flow rate of Panchganga river basin was estimated 725.26 m3/s in 2003.

The crux of this scene is likely to be due to lower and erratic rainfall in this hilly tract during that time. Less rain also means lower river flows, which means less discharge as well. Additionally, human activities within the river system (e.g. irrigation or urban water supply drainage) may also influence water discharge in the Panchganga river basin. Also, rivers become overtrained more, and flow rates are even lower and more frequent low discharge periods are lengthened, especially in droughts, also at periods of high water need; this disturbs naturally the usual rainy situation in the basin, that leads to more “dry weather”.

The discharge rate showed moderate fluctuations between 1979 and 1984, with a slight rise and fall but no extreme peaks, indicating a relatively stable period (Fig. 5). In 1985 and 1986, discharge declined, possibly due to reduced rainfall or better water retention in the catchment. A noticeable increase occurred in 1987 and 1988, followed by a slight decrease in 1989. Subsequently, discharge values increased significantly from 1990 to 1991 and peaked in 1994, suggesting high rainfall or heavy releases from reservoirs. This was followed by a decline in 1995, with minor fluctuations in 1996 and 1997, the year 1997 showing one of the highest peaks (3590 m^3^/s), indicating an extreme flood event. From 1998 to 2003, the discharge trend decreased significantly, with 2003 recording the lowest discharge (725.26 m^3^/s) in this entire dataset, possibly due to drought or regulated water management. After 2004, discharge values showed a sharp upward trend, peaking in 2005 (3340 m^3^/s) and again in 2006, followed by fluctuations in subsequent years. In recent years, particularly in 2019 (4787.89 m^3^/s) and 2021 (4100 m^3^/s), the discharge rates showed extreme peaks, indicating increasingly severe flood occurrences likely due to climate change, intense rainfall, and catchment changes.

Because of the severe probability of flooding and the devastating effects it can have on populations’ public safety, critical infrastructure, and economic sustenance, Panchganga river villages are particularly at risk. Loss of access to vital services, destruction of homes and agricultural land, and displacement are all possible outcomes of flooding. GEV and LPT-III approaches have been found to be useful in predicting floods in the Panchganga river basin as they have reliable capability to predict extreme floods and help improve early warning and risk issues. However, low availability of hydrological data, in particular the discharge and wet water levels (WL) in the Indian river basins, restricts the application range of the FFA. In addition, as the Krishna river system is an international river, it is categorised and the real-time data is not available in the public domain, for flood assessment. The results of this study are important for flood prevention, such as levee construction, improvement of drainage system, establishment of timely-warning measures. In addition, community participation and disaster prevention are key to reducing flood impact, and the coordinated reaction in a disaster of this nature.

### 3-Parameter loglogistic distribution

**Fig. 6:**
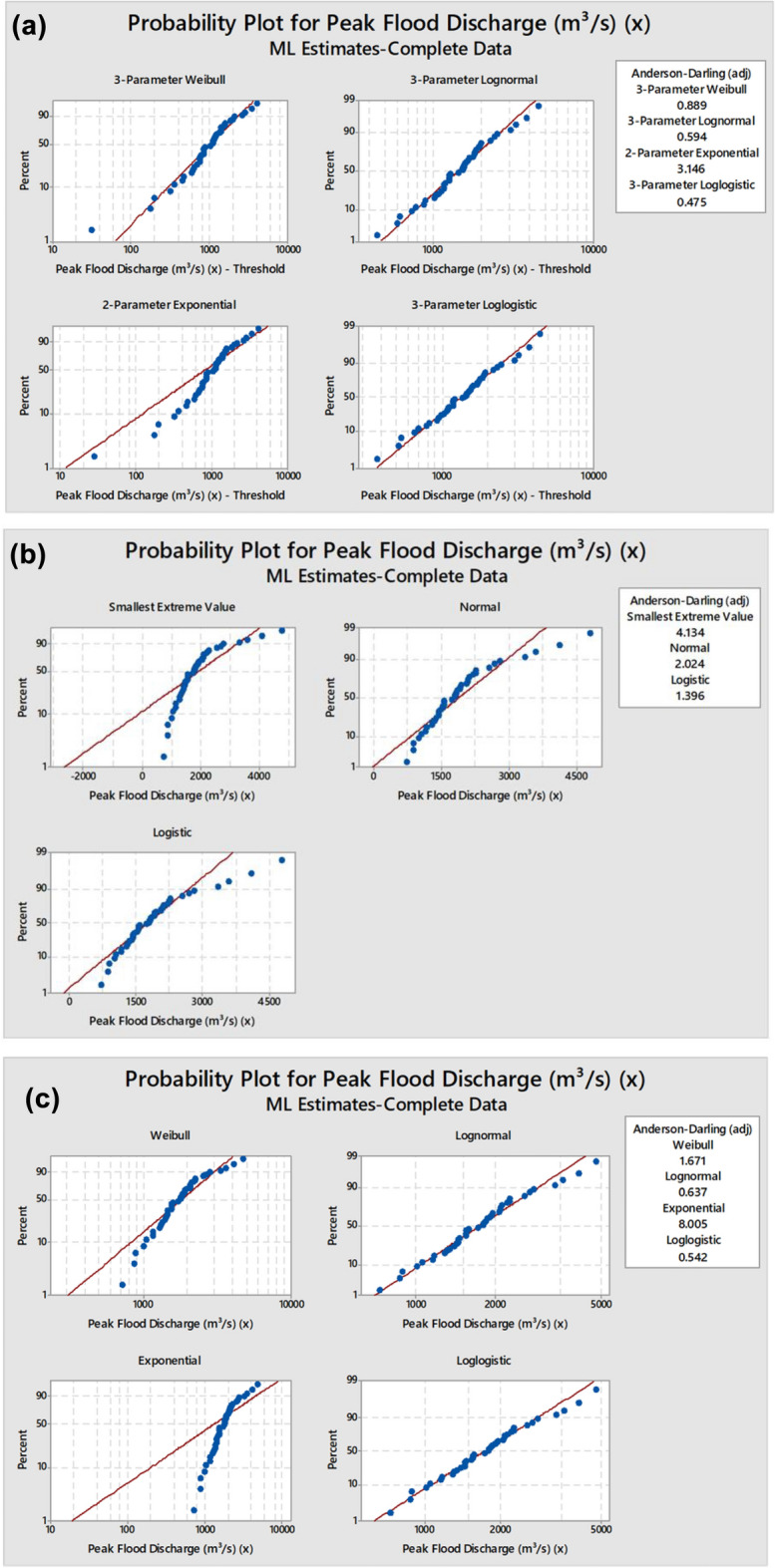
(**a**) Best fit probability distribution results based on FFA. (**b**) Best fit probability distribution results based on FFA. (**c**) Best fit probability distribution results based on FFA.

In this analysis we compare different statistical distribution to determine which best fit a given dataset. This comparison is based on Anderson darling test statistics value and parameters are estimated by using maximum likelihood estimators. From the Fig it can be inferred that the 3-parameter logistic distribution is the best most suitable probability distribution among all distribution based on Anderson darling test statistic value (Fig. 6).

In this table the flood discharge analysis results are obtained. For our data 3-parameter logistic distribution is recommended as the best fitted probability distribution for estimating flood percentile for different periods. The 3-Parameter Loglogistic distribution shows the lowest Anderson–Darling statistic (0.475) and therefore provides the best fit for this dataset.

These percentiles could be potentially be employed in the the implementation of flood discharge efforts (Table 6).

**Table 6:** Goodness-of-Fit (Anderson–Darling Test Statistics).

Distribution	Anderson–Darling (adj) Value
Weibull	1.671
Lognormal	0.637
Exponential	8.005
Loglogistic	0.542
3-Parameter Weibull	0.889
3-Parameter Lognormal	0.594
2-Parameter Exponential	3.146
3-Parameter Loglogistic	**0.475**
Smallest Extreme Value	4.134
Normal	2.024
Logistic	1.396
Exponential	8.005
Loglogistic	0.542
3-Parameter Weibull	0.889
3-Parameter Lognormal	0.594
2-Parameter Exponential	3.146
3-Parameter Loglogistic	**0.475**

Bold values indicate the lowest Anderson–Darling statistic and the best-fi tting distribution.

### Winters’ method using forecast for peak flood discharge (m^3^/s) (x)

To model and forecast annual peak flood discharge, the Winters’ Exponential Smoothing Method (Multiplicative) was employed, utilizing a time series dataset spanning 43 years (1979–2021). This method effectively captures three key components of a time series: level (α), which represents the smoothed mean of the series; trend (γ), indicating the long-term directional movement; and seasonality (δ), which accounts for cyclical fluctuations. The smoothing constants selected for this study were α = 0.1, γ = 0.1, and δ = 0.1, reflecting a modest sensitivity to recent changes in the data. The model was validated using standard accuracy metrics including Mean Absolute Percentage Error (MAPE), Mean Absolute Deviation (MAD), and Mean Squared Deviation (MSD), which yielded values of 38, 610, and 694,572 respectively. These results suggest a moderate level of forecasting precision suitable for planning purposes.

To model and forecast annual peak flood discharge, the Winters’ Exponential Smoothing Method (Multiplicative) was employed, utilizing a time series dataset spanning 43 years (1979–2021). This method effectively captures three key components of a time series: level (α), which represents the smoothed mean of the series; trend (γ), indicating the long-term directional movement; and seasonality (δ), which accounts for cyclical fluctuations. The smoothing constants selected for this study were α = 0.1, γ = 0.1, and δ = 0.1, reflecting a modest sensitivity to recent changes in the data. The model was validated using standard accuracy metrics including Mean Absolute Percentage Error (MAPE), Mean Absolute Deviation (MAD), and Mean Squared Deviation (MSD), which yielded values of 38, 610, and 694,572 respectively. These results suggest a moderate level of forecasting precision suitable for planning purposes (Table 7).

**Table 7:** Winters’ Method for Peak Flood Discharge.

Time	Peak flood discharge (mÂ^3^/s)	Smooth	Predict	Error
1979	870	932.97	986.79	− 116.79
1980	1917.6	1309.3	1380	537.6
1981	1790	1588.37	1672.65	117.35
1982	1430.6	1859.23	1953.55	− 522.95
1983	1545	1958.53	2050.31	− 505.31
1984	1795	1675.52	1748.18	46.82
1985	1570	2384.49	2483.97	− 913.97
1986	2050	1389.86	1442.34	607.66
1987	1392	1525.37	1584.8	− 192.8
1988	2080	1344.67	1394.05	685.95
1989	2205	1905.74	1979.02	225.98
1990	2250	2008.09	2083.87	166.13
1991	2553	1628.46	1688.5	864.5
1992	1306	2490.53	2586.91	− 1280.91
1993	1447	2651.58	2741.7	− 1294.7
1994	2680	2794.48	2877.04	− 197.04
1995	1170	2949.5	3032.69	− 1862.69
1996	1900	2446.25	2501.73	− 601.73
1997	3590	3199.47	3264.3	325.7
1998	1051	2055.53	2097.97	− 1046.97
1999	1540	1930.16	1961.12	− 421.12
2000	890	1737.12	1761.33	− 871.33
2001	1150	2114.31	2133.89	− 983.89
2002	1443	2035.93	2045.67	− 602.67
2003	725.26	1595.35	1598.34	− 873.08
2004	1832	1935.94	1928.58	− 96.58
2005	3340	2074.3	2065.3	1274.7
2006	2796.56	2440.04	2444.21	352.35
2007	1820.56	2423.73	2431.25	− 610.69
2008	1951.57	2116.43	2117.69	− 166.12
2009	1344.51	2840.79	2840.25	− 1495.74
2010	1463.83	1584.19	1575.06	− 111.23
2011	1720.64	1558.16	1547.97	172.67
2012	1010.61	1377.72	1370.26	− 359.65
2013	1854.07	1690.95	1676.94	177.13
2014	1534.09	1723.69	1711.24	− 177.15
2015	1285.65	1326.29	1315.15	− 29.51
2016	2267.37	1765.39	1750.01	517.36
2017	1542.07	2058.86	2047.2	− 505.13
2018	2072.09	2138.39	2120.49	− 48.4
2019	4787.89	1994.63	1977.3	2810.59
2020	2117	2049.77	2059.55	57.45
2021	4100	2671.71	2685.11	1414

The model demonstrated a strong ability to follow the historical patterns of peak flood discharge with reasonable predictive capacity. Actual and predicted values from 1979 to 2021 showed generally close agreement, although some years exhibited larger residuals, particularly during high-discharge events such as 2019 and 2021. The residual plots indicated that the errors were normally distributed with constant variance and no significant autocorrelation, supporting the reliability of the smoothing model (Table 8).

**Table 8:** Winters’ method using forecast peak flood discharge.

Year	Forecast	Lower	Upper
2022	1757.61	262.4	3252.82
2023	1797.08	296.12	3298.03
2024	1542.85	35.8	3049.9
2025	2041.86	528.36	3555.36
2026	2057.23	536.93	3577.54
2027	1638.23	110.78	3165.68
2028	2286.41	751.47	3821.35
2029	2511.59	968.83	4054.35
2030	2771.66	1220.75	4322.58
2031	2937.9	1378.5	4497.29
2032	2425.3	857.1	3993.49
2033	3299.36	1722.06	4876.66
2034	1961.15	374.43	3547.88
2035	2003.2	406.75	3599.65
2036	1718.14	111.67	3324.61

Future forecasts generated for the years 2022 to 2036 indicate varying magnitudes of peak discharge, with the highest values predicted in 2031 (2937.9 m^3^/s) and 2033 (3299.36 m^3^/s), suggesting possible extreme flood scenarios in those years. The prediction intervals, ranging from lower bounds (e.g., 35.8 m^3^/s in 2024) to upper bounds (e.g., 4876.66 m^3^/s in 2033), provide a robust range for uncertainty assessment in flood risk planning. This forecasted dataset serves as a valuable decision-support tool for hydrologists, disaster managers, and urban planners for designing flood mitigation infrastructure, allocating resources, and developing early warning systems.

In conclusion, the Winters’ Exponential Smoothing method proves to be an effective approach for capturing seasonal and trend components in hydrological time series, offering reliable insights into future flood discharge variability, which is critical for adaptive water resource management in the context of climate variability and urban expansion.

### Winters’ method plot for peak flood discharge (m^3^/s) (x)

The graph visually represents the application of the Winters’ Multiplicative Exponential Smoothing method for forecasting peak flood discharge. The blue line indicates the actual observed values, showing noticeable year-to-year variability and occasional extreme peaks. The red squares denote the fitted values generated by the model for the historical period, which closely follow the actual trend, demonstrating a good fit.

The green dashed line with diamonds shows the forecasted peak flood discharges from 2022 onward. These forecast values indicate a general upward trend in flood magnitude, highlighting a possible increase in future flood severity. Accompanying these forecasts, the purple triangles represent the 95% prediction intervals, which widen progressively with time. This widening reflects increasing uncertainty in the long-term forecast.

**Fig. 7:**
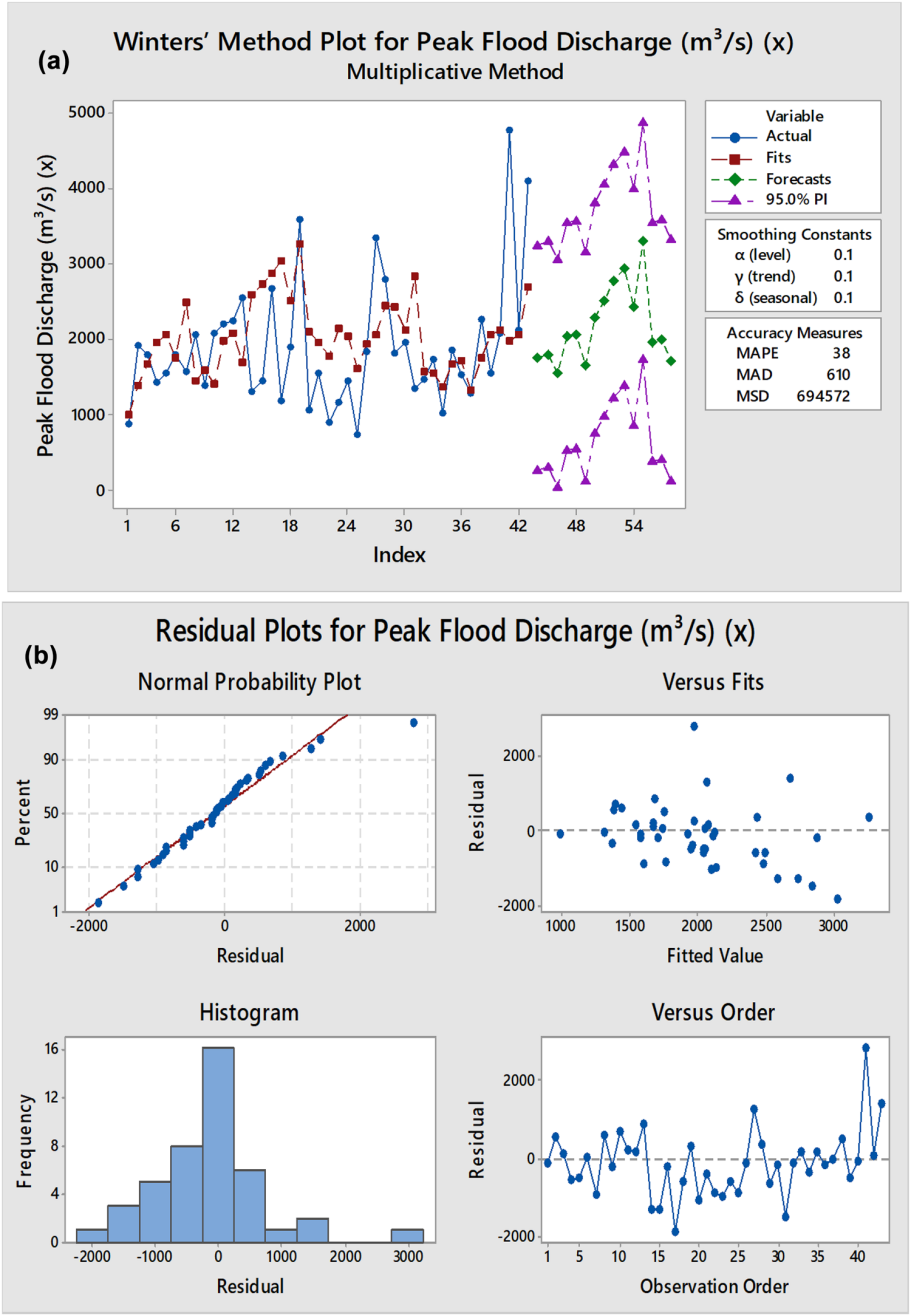
(**a**) Winters’ Method plot for peak flood discharge graph. (**b**) Winters’ Method plot for peak flood discharge graph.

Overall, the graph suggests that the model effectively captures historical patterns and provides a reasonable projection for future flood events, with uncertainty appropriately visualized through prediction intervals (Fig. 7).

The residual plots indicate that the model errors are approximately normally distributed, as shown in the Normal Probability Plot. The Histogram further supports this by clustering residuals around zero. The Versus Fits plot shows no clear pattern, suggesting constant variance. The Versus Order plot reveals random scatter, indicating that the residuals are uncorrelated over time.

### Limitations of the study

The present study is subject to certain limitations. The analysis is based on historical annual maximum discharge data obtained from the Central Water Commission and Maharashtra Water Resources Department for the period 1979–2021, and real-time high-resolution hydrological data were not available. One of the major limitations of the study was that real-time recent hydrological data was not available, which could have improved the accuracy of the analysis. The Panchganga river is ungauged, but the restricted availability of high-resolution data to the general public inhibits an assessment of modern flood characteristics and changes in flood magnitudes. Moreover, application of GEV and LPT-III models to simulate the future inundation also provides a useful past perspective while representing only part of current hydrologic processes, affected by accelerated urban expansion, climate change, and land-use modifications. There is great potential for better flood risk predictions and planning more resilient approaches to flood management if consistent and up-to-date hydrological information were available.

## Conclusions

FFA was conducted for the Panchganga river basin with the assistance of 43-year peak discharge record in an attempt to forecast the future magnitude of flood using GEV and LPT-III approach. An extensive distribution of maximum discharge volumes related to flood events in the basin, varying from the maximum observed discharge of 4787.89 m^3^/s in 2019 to the minimum discharge of 725.26 m^3^/s in 2003, was obtained from the analysis. These changes are induced by monsoonal rainfall input, land use modifications, and the regulation operation at upstream reservoirs. Using probability distributions and hydrograph analysis, it is established that flood occurrences are non-linear in nature: smaller, less severe events are common, whereas larger, more severe floods are less frequent but inconsequential. An expected flood relationship provided by the Gumbel distribution was y = 6295.6(x) + 1333.9 with R^2^ = 0.8403 indicating that higher flood magnitudes were associated with higher return periods.This research offers valuable insights for policymakers and environmental planners, aiding in the development of effective flood management strategies, risk mitigation efforts, and long-term sustainability planning for the Panchganga river basin. Future research should focus on non-stationary flood frequency analysis incorporating climate change effects, land-use dynamics, and reservoir operations. Comparative evaluation of GEV and machine-learning-based flood models using high-resolution rainfall and remote sensing data would further improve flood prediction accuracy. Integration of hybrid statistical–ML frameworks is recommended for developing resilient flood forecasting systems in Indian river basins.

## Data Availability

The datasets used and analyzed during the current study are available from the corresponding author on reasonable request.
